# The DSM needs more than revision: five blind spots and a case for dialogical redesign

**DOI:** 10.1017/S0033291726104644

**Published:** 2026-05-14

**Authors:** Lars Veldmeijer, Laura Batstra, Jim Van Os

**Affiliations:** 1 Division Neuroscience, Utrecht University Medical Center, Utrecht, Netherlands; 2 University of Groningen: Rijksuniversiteit Groningen, Netherlands

**Keywords:** classification, diagnosis, DSM, public mental health, scientific inference, lived experience, epistemic governance, identity intervention, design, dialogue

## Abstract

**Background:**

Recent proposals for revising the Diagnostic and Statistical Manual of Mental Disorders (DSM) aim to improve psychiatric diagnosis. While these efforts reflect substantial ambition, they continue to operate within assumptions embedded in the DSM’s underlying classificatory logic. This editorial examines whether such incremental revision is sufficient.

**Methods:**

We provide a critical analysis of the recently published DSM roadmap and accompanying subcommittee commentaries. Drawing on contemporary literature, we identify five structural blind spots in the current reform agenda: public mental health, scientific inference, lived experience, epistemic governance, and the function of diagnosis. Based on this analysis, we propose an alternative dialogical redesign for the DSM.

**Results:**

We argue that current revision considerations risk increasing complexity without resolving fundamental limitations in psychiatric classification. Specifically, our analysis highlights several areas that warrant further consideration, including the relationship between diagnostic expansion and societal conditions, the applicability of group-level scientific findings to individual care, the incorporation of experiential knowledge, participatory governance in revision processes, and the identity-related implications of diagnosis. In response, we propose redesigning the DSM as a hybrid dialogical system that retains coarse-grained classificatory categories for pragmatic purposes while shifting diagnostic practice toward contextual interpretation, collaborative meaning-making, relational understanding, and individualized care formulation.

**Conclusions:**

The challenges facing psychiatric diagnosis require more than incremental refinement. We therefore argue for a dialogical redesign of the DSM that better reflects the context-dependent, experiential, and relational nature of mental health conditions, positioning diagnosis as a starting point for collaborative inquiry.

## Background

This editorial offers a critical ‘diagnosis’ of the recently published roadmap (Oquendo et al., 2026) and subcommittee commentaries for the future of the DSM (Cuthbert et al., 2026; Drexler et al., 2026; Öngür et al., 2026; Wainberg et al., 2026), and advances an alternative way forward that integrates their respective strengths within a different conceptual framework. First, we argue that, despite its reformist language, the roadmap remains marked by a series of profound blind spots. These blind spots are significant, not only conceptually but also because the DSM remains the structural backbone of mental health care in high-income nations. Therefore, the question is not whether the DSM can be refined, but rather, whether its underlying logic is still fit for purpose in the context of a deepening mental health care crisis (Aftab, 2026; Fried, 2026; Tandon, 2026; Van Os, 2026). In our view, the current considerations for the future of DSM show great ambition but also risk adding further complexity to the same fragile design. We aim to illustrate this by analyzing five blind spots, i.e. public mental health, scientific inference, lived experience, epistemic governance, and the function of diagnosis, showing that reforming the DSM without addressing its core assumptions risks perpetuating the very problems it seeks to solve. Second, we propose redesigning the DSM as a hybrid dialogical system that helps with explaining and understanding.

While some of the limitations discussed in this editorial, such as heterogeneity and limited prognostic precision, are not unique to psychiatry, they are arguably amplified in this field. A key reason is that psychiatric diagnosis concerns phenomena that are primarily experiential in nature, whereas the scientific frameworks used to study them are largely developed within a paradigm oriented toward measurable physical processes. This creates a distinctive epistemic tension as psychiatry seeks to classify and explain phenomena that are not directly accessible within the same domain in which its explanatory models are formulated. In this editorial, we refer to mental disorders as mental health conditions, as recommended by the Lancet Commission report on Ending Stigma and Discrimination in Mental Health (Thornicroft et al., 2022). According to DSM-5 (APA, 2013), mental health conditions are usually associated with significant distress or disability in social, occupational, or other important activities.

## Blind spot 1: Public mental health

The first blindness concerns public mental health. Across high-income countries, DSM-based systems are estimated to classify roughly 25% of the population as mentally disordered annually, with especially steep increases among adolescents and young adults (Have et al., 2023; Wang et al., 2025). At the same time, specialist mental health services in various Western countries have become structurally inaccessible, with long waiting lists and rationed care (Department of Health and Social Care, 2026; McGorry et al., 2025; Van Os & Guloksuz, 2023). Yet the DSM roadmap does not ask whether this situation reflects a failure of the diagnostic model itself. Instead, rising prevalence is implicitly interpreted as rising pathology in individuals rather than as a potential signal of worsening collective conditions (Kirkbride et al., 2024), diagnostic inflation (Batstra & Frances, 2012), or overdiagnosis (Kazda et al., 2021; Rødgaard et al., 2019). Large-scale epidemiological data show that deteriorating mental health indicators among young people track social inequality, educational pressure, sociopolitical polarization, housing insecurity, loneliness, climate anxiety, and an increasingly extractive attention economy. From a ‘push’ perspective, these indicators are more plausibly understood as population-level stressors than as evidence of expanding individual disorders (Van Os & Guloksuz, 2023).

From a ‘pull’ perspective, it has been hypothesized that individuals increasingly interpret and report milder forms of distress as mental health problems (Foulkes & Andrews, 2023); that diagnostic categories may undergo conceptual expansion in digital environments, where portrayals of conditions such as ADHD extend beyond clinical criteria while being widely recognized and validated by viewers (de Vries, Batstra, & Van Assen, 2025); and that, in some contexts, diagnostic labels (e.g. ASD and ADHD) are actively sought and valued because they can confer tangible benefits, such as disability accommodations and sickness benefits (Davidovitch, Shmueli, Rotem, & Bloch, 2021; Werkhoven, Anderson, & Robeyns, 2022). These trends may equally contribute to diagnostic expansion, as they reflect and promote help-seeking behavior and cultural valuation of psychiatric identity. Instead of critically examining and analyzing the role of the current diagnostic model and its underlying logic in this push and pull mechanism, the DSM’s taskforce response remains strongly oriented toward ever more precise individual classification.

## Blind spot 2: Scientific inference

The second blindness concerns scientific inference. After more than 50 years of research, psychiatry has produced an abundance of group-level associations linking mental health conditions to genetics, neurobiology, immune markers, trauma exposure, and environmental adversity. Yet associations observed between groups cannot readily be translated into within-person processes (Fisher, Medaglia, & Jeronimus, 2018). This limitation is reinforced by the heterogeneity and context dependence of mental variation, which unfolds dynamically across time through changing psychological states and situated behavior (Van Os et al., 2021, 2023). As a result, many group-level findings remain imprecise, weakly replicable, and non-specific, explaining little variance at the level where care takes place: the unique person seeking help (Van Os et al., 2019; Veldmeijer et al., 2025). There are no robust biomarkers – acknowledged by the Biomarkers and Biological Factors Subcommittee (Cuthbert et al., 2026) – no clear boundaries between diagnostic categories, and no reliable prediction of prognosis or treatment response based on DSM labels.

In contrast to many areas of somatic medicine, the phenomena that psychiatric diagnosis seeks to describe, i.e. subjective experiences, meanings, and relational dynamics, are not directly observable within the same measurable physical domain as their proposed biological correlates. Instead, diagnosis relies on indirect proxies, such as self-report and observed behavior, interpreted within clinical and cultural frameworks. This contributes to a form of heterogeneity that is not only quantitative but also qualitative, as category boundaries are less constrained by independently verifiable markers. The roadmap’s efforts to integrate more biological (Cuthbert et al., 2026) and contextual data (Wainberg et al., 2026), as well as to incorporate functioning and quality-of-life elements (Drexler et al., 2026), are commendable but do not resolve this fundamental limitation without a redefinition of its underlying logic. On the contrary, it risks multiplying complexity without increasing usefulness, by embedding ever more extremely weak, imprecise, and variable group-level signals into individual diagnostic reasoning. What is presented as scientific progress may therefore amount to refining a framework that empirical evidence has already shown to be intrinsically limited (Van Os, 2026).

## Blind spot 3: Lived experience

A third blindness concerns lived experience. The persistent, albeit implicit, biomarker-oriented logic underlying DSM psychopathology reflects two intertwined tendencies: the marginalization of experiential knowledge, and psychiatry’s recurrent preoccupation with its own conceptual frameworks (Van Os, 2017). Yet mental health conditions are inherently multidimensional (Haslam, McGrath, Viechtbauer, & Kuppens, 2020) shaped by relationships, meaning, and social conditions (Bracken & Thomas, 2005). Rather than residing within isolated individuals, it unfolds across biological, psychological, social, relational, and existential domains that demand interdisciplinary understanding (Leucht et al., 2025). People with mental health conditions and mental health professionals alike report that mental health conditions are not well captured by symptom lists or categorical labels (Kohne, De Graauw, Maas, & Van Os, 2023). People describe experiences of entrapment, narrowing of consciousness, loss of future, and the emergence of powerful, intrusive inner dynamics that feel dialogical, coercive, and difficult to escape. Across diagnostic categories, these experiential patterns show striking similarities. What appears to matter most for relief is not diagnostic specificity but relational safety, social holding, and opportunities to regain agency through learning, experimentation, and meaning-making. Although the Structure and Dimensions Subcommittee explores dimensionality as a promising direction (Öngür et al., 2026), the DSM plans remain largely silent on how phenomenology is to be meaningfully integrated into the diagnostic framework.

## Blind spot 4: Epistemic governance

The fourth blindness is epistemic governance. Although the roadmap emphasizes the involvement of people with lived experience, this participation remains – based on the limited information that the authors have shared on this topic (Oquendo et al., 2026) – largely consultative rather than constitutive. The current summary and commentaries do not specify how lived experience will shape the design process or the framework itself. Indeed, given the significant disagreement and inconclusiveness in the field of mental health care, lived experience involvement is crucial for meaningful transformation (Beresford, 2026; Fellowes, 2023; Friesen, 2024; Jones et al., 2021; Tekin, 2022). Lived experience can reveal dimensions of distress, such as phenomenological insight (Ritunnano et al., 2026), that otherwise remain overlooked or underappreciated (Dings & Strijbos, 2025). However, as the plans currently indicate, the DSM continues to be designed primarily by scientific experts, within predefined assumptions about what counts as evidence and validity, reproducing a familiar pattern, largely consistent with the design processes of earlier editions and similar innovation processes (Tekin, 2022; Veldmeijer et al., 2024; Veldmeijer & Van Os, 2025).

As psychiatry still lacks a clear account of what constitutes mental health conditions, it is imperative that diverse groups of people with lived experience take on an architectural role in shaping the DSM from the bottom up (Veldmeijer et al., 2024). Although this does not resolve the long-standing criticism of the DSM being formally expert-led, such epistemic governance is diversified through including people with lived experience as experiential experts. If the taskforce genuinely aims to innovate and challenge basic implicit assumptions underlying DSM’s conceptualization, which have always been influenced by ‘collective emotions’ (Brencio, 2026), dissensus should be the source of new ideas and diverse perspectives should be seen as an asset (Speyer & Ustrup, 2025). Taken together, the evidence suggests that meaningful lived experience involvement is nearly 75 years overdue (Gagné-Julien & Friesen, 2026).

## Blind spot 5: The function of diagnosis

A fifth, and crucial, blindness concerns the function of diagnosis itself. In medicine, diagnoses are tools that serve specific purposes: they – more or less – inform prognosis and guide care needs. A key issue underlying the limited utility of psychiatric diagnosis is that it attempts to provide a fine-grained classificatory structure for phenomena that are not directly measurable in the same domain as their proposed explanatory mechanisms. As a result, diagnostic categories are necessarily constructed at a level of abstraction that provides orientation but only limited precision at the level of the individual. In much of somatic medicine, diagnostic processes operate within a shared physical domain in which both indicators (e.g. biomarkers, imaging findings) and disease processes can be measured and iteratively refined. Again, in psychiatry, the core phenomena – subjective experiences, meanings, and relational dynamics – are not directly measurable in this domain, even though diagnostic systems partially rely on measurable proxies.

This helps explain why DSM classifications offer, at best, coarse guidance in relation to prognosis and care needs and why their function in clinical practice often diverges from their intended role. People with the same diagnosis of depression, psychosis, or autism can therefore have radically different trajectories, risks, resources, and forms of support that matter most (Van Os et al., 2021). Thus, mental health practice centers on responding to each individual’s lived realities and capacities, yet none of this is central to the DSM framework. A diagnosis should also be useful for people with mental health conditions and provide usable information within the context of their lives, not only for researchers and mental health professionals. The weak relationship between care needs and DSM classifications, combined with the distance between the DSM and the narratives of people seeking help, constitutes the weakest point of the DSM, which is not improved by the proposed changes.

## The risk of identity intervention

Instead of informing prognosis and guiding care needs, DSM diagnosis functions as something else: a potential identity intervention (Van Os, 2026; Veldmeijer & Van Os, 2026). Particularly in children and young people, being told that one has a disorder ‘in the head’ shapes self-concept, expectations, and life narratives (te Meerman, Freedman, & Batstra, 2022). The DSM roadmap summary acknowledges that this reification problem extends across mental health professionals, people seeking help, and the broader public (Oquendo et al., 2026). Although mental health professionals (should) emphasize that diagnoses in the DSM are descriptive and do not have explanatory power by design (APA, 2013), they are commonly interpreted as statements about who someone is and what is wrong with them. While some people experience this as helpful, it can also encourage self-stigma, reduce perceived agency, capture people’s rich values, and narrow imagined futures (Batstra & Timimi, 2024; Levinovitz & Aftab, 2025; O’Connor, Kadianaki, Maunder, & McNicholas, 2018; Tekin, 2025; Van Eck et al., 2025; Veldmeijer, Terlouw, Boonstra, & Van Os, 2025a; Werkhoven et al., 2022). This requires revision efforts that extend beyond scientific validity and clinical reliability to examine whether diagnostic classifications facilitate meaningful dialogue as non-essentialist narratives. This is crucial because subjective experience and meaning are central to understanding mental phenomena and, as argued above, may be constrained by the imposition of diagnostic labels (Broeker & Arnaud, 2024; De Boer, 2026; Veldmeijer et al., 2024). Even when a diagnosis is experienced as beneficial, there can still be downstream negative consequences, not only for the individual but also on the societal level, such as rising sickness absence and disability-related costs associated with expanded medicalization (Beeker et al., 2021; Conrad, Mackie, & Mehrotra, 2010). The trade-offs are therefore not fully captured by the fact that some people find validation in DSM diagnoses.

## Redesigning the DSM as a potential conversation piece?

As discussed in this editorial, psychiatry represents an intensified and qualitatively distinct instance of broader challenges in medicine. This difference has important implications for how diagnostic systems should be conceptualized and used. It requires a framework that captures a more complete picture of how mental health conditions shape a person’s life, including both its positive and negative aspects (Daly & Gallagher, 2019; Sartorius, 2015). To counter the blind spots of current considerations, we argue for the subcommittees to combine their strengths toward a more dialogical and person-centered use of diagnosis.

We acknowledge that exclusively dialogue-focused approaches introduce new challenges, including the risk of overstating individual experiences, variability between mental health professionals, and difficulties in standardization for service organization, research, and policy. A more feasible direction therefore lies in developing hybrid models that retain the pragmatic functions of classification frameworks (e.g. for access, entitlements, commissioning, epidemiology, and service planning), while expanding space for contextual, experiential, and care-oriented formulations in diagnostic practice. Our solution is therefore not to abolish classification altogether but to make it broader. This could involve maintaining the DSM at a coarse-grained level of classification (e.g. depression, autism, psychosis, anxiety, OCD, PTSD, and so forth), while moving away from increasingly fine-grained diagnostic subcategories that often lack empirical robustness in light of the substantial heterogeneity within groups. Cross-national comparisons show, for example, that the prevalence of diagnostic classifications vary markedly between geographically proximate countries, with variation largely driven by contextual and systemic factors (De Veen et al., 2026). This hybrid design is also more compatible with potentially valuable subcommittee initiatives, such as incorporating socioeconomic, cultural, and environmental determinants and intersectionality (Wainberg et al., 2026). Broader categories, redesigned in co-creation with people with lived experience, could preserve their pragmatic value for communication, service organization, and initial clinical orientation, while reducing the risk of reifying narrowly defined diagnostic entities.

Reconsidering the DSM in this way implies redefining the focus and function of DSM-based knowledge. Here, we introduce and build on Karl Jaspers’ (1913) epistemological distinction, adopted from Wilhelm Dithley (Gough, 2023), between *Erklären* and *Verstehen.* In broad strokes, *Erklären* refers to causal, natural-scientific explanation of the objective outer world, whereas *Verstehen* concerns the interpretative understanding of lived experience, the subjective inner world. These correspond to two irreducible yet complementary questions that remain relevant for the underlying logic of psychiatric diagnostic frameworks, i.e. what causes a condition, and how is the condition meaningful within the person’s life. For Jaspers, certain phenomena were considered not meaningfully understandable. So-called primary delusions were regarded as ‘ununderstandable’ and should therefore fall outside the scope of *Verstehen.* However, this position requires careful reconsideration considering contemporary findings. For example, recent phenomenological research suggests that delusional experiences may exhibit forms of intelligibility when situated within a person’s experiential and affective context (Feyaerts et al., 2021; Ritunnano, 2022; Ritunnano et al., 2022; Ritunnano et al., 2026). Moreover, we also advance the normative claim that every person seeking or needing help is first and foremost a human being who warrants an attempt at understanding, even where intelligibility is not immediately apparent from the clinical perspective (Henriksen, 2013; Pienkos, 2024; Ritunnano, 2022; Veldmeijer et al., 2025b).

We further propose broadening Jaspers’ original notion of *Erklären* beyond biological mechanisms to include contextual, social, and relational contributors to psychopathology. However, even such an expanded explanatory framework cannot replace first-person meaning-making, which remains an inherently interpersonal and dialogical act within clinical encounters (Hoff, Maatz, & Vetter, 2020). In short, any attempt at understanding the subjective inner world – how conditions are lived – and how this experience relates to contextual factors and relational dynamics in a person’s life requires dialogue and collaborative sense-making (De Haan, 2020; Scheepers, 2021; Tekin, 2025; Veldmeijer et al., 2024; De Rooy et al., 2025). Expanded explanatory frameworks may provide early orientation for intervention (*Erklären*), but they cannot yield definitive understanding of the individual case (*Verstehen*).

From this starting point, practical diagnostic work can shift toward a more individualized process, focusing on the person’s unique configuration of experiences, vulnerabilities, context, and care needs. This may involve examining the unique ‘problem-sustaining patterns’ of individuals seeking help (Voerman et al., 2025). To promote a more comprehensive understanding, we suggest expanding the DSM beyond individual-level difficulties by incorporating domains for context diagnosis, enabling qualitative assessments of which contextual factors in an individual’s life need addressing to reduce suffering and enable flourishing (Batstra & Frances, 2025). This may prevent the systematic decontextualization of mental health conditions (De Ridder & Van Hulst, 2023). In this way, broad categories can function as provisional entry points for exploration rather than endpoints of diagnostic reasoning. Such a redesign could be probabilistically informative, enabling professionals to distinguish between conditions while providing sufficient structure for clinical encounters without being overly deterministic. Until better alternatives are widely available for adoption, the DSM can serve as a meta-framework and ‘conversation piece’, both among professionals and researchers as well as between professionals and people seeking help (Hoff et al., 2020; Terlouw et al., 2022; Veldmeijer et al., 2024). A conversation piece refers to the DSM as a tool that structures and mediates dialogue between stakeholders (Veldmeijer et al., 2024). In this sense, it may function as a boundary object. A boundary object is an artefact that is sufficiently flexible to accommodate multiple perspectives and activity systems (e.g., professionals, people seeking help, researchers), while remaining robust enough to enable coordination and shared reference across these domains (See Terlouw et al., (2022) for a more comprehensive explanation of the potential of boundary objects as dialogical learning accelerators).

A final advantage of such a coarse-grained approach is that it may mitigate the function of diagnosis as an identity intervention. Hence, broader and less specific categories are less likely to be interpreted as definitive statements about who a person is, thereby reducing the likelihood that diagnoses are internalized as fixed identities, and preserving space for negotiated meanings and individualized care trajectories (Van Os et al., 2021).

## Conclusion

We analyzed the summary and commentaries for the future DSM and conclude that they show ambition but remain structurally misaligned with the phenomena they aim to capture. These limitations reflect a deeper mismatch between subjective, context-dependent phenomena, and a framework oriented toward fine-grained categorical description. Further refinement within this same logic risks increasing complexity without improving usefulness. We therefore propose a functional redefinition that better fits psychiatry as an intensified and qualitatively distinct instance of broader challenges in medicine. According to our analysis, a potential path forward is to redesign the DSM as a hybrid, dialogical system that retains coarse-grained categories for orientation and probabilistic information, while shifting diagnostic practice toward contextual interpretation, meaning-making, relational dynamics, and care needs in clinical practice. In this concept, classification functions as a starting point rather than an endpoint. Reconsidering the DSM in this way does not resolve all challenges, but it could realign diagnosis with the realities that mental health care faces.
